# Epidemiology and complications of rheumatoid arthritis in the Indigenous Australian population

**DOI:** 10.1007/s00296-025-05951-y

**Published:** 2025-08-07

**Authors:** Johannes C. Nossent, Helen I. Keen, David B. Preen, Charles A. Inderjeeth

**Affiliations:** 1 Rheumatology Group, School of Medicine, University Western Australia, 35 Stirling Highway (M503), Perth, WA Australia; 2 Department of Rheumatology, Fiona Stanley Hospital, Perth, WA Australia; 3 School of Population and Global Health, University Western Australia, Perth, Australia; 4 Sir Charles Gairdner Osborne Park Health Care Group, Perth, WA Australia

**Keywords:** Rheumatoid arthritis, Indigenous, Incidence, Prevalence, Morbidity, Mortality

## Abstract

Rheumatoid Arthritis (RA) can present differently in Indigenous populations. Data on RA in Indigenous Australians (IA) are almost non-existent. A population-based cohort study of disease incidence (IR) and point prevalence (PR) rates per 100.000 and clinical outcomes in IA patients hospitalised with RA compared to non-Indigenous (NI) patients with RA using longitudinal administrative health data over 30 years. For IA patients (*n* = 282) the RA IR was 23.52 (CI 14.99–35.14) which did not differ from the IR in NI patients 27.85 (CI 18.48–40.29) (*p* = 0.67) with the 2015-point PR in IA at 373.5 (CI 336.5-413.3) versus 375.3 (CI 338.3–145.2) in the NI population (*p* = 0.91). At index admission, IA patients with RA were younger, more often smokers with a higher prevalence of diabetes mellitus and obesity. Over the disease course, IA patients had lower readmission and ED visits rates for RA, were less likely to undergo joint surgery and had lower rates of confirmed osteoporosis, despite higher fracture rates. In contrast, extraarticular RA manifestations were as frequent in both groups. The age adjusted standardised mortality rate ratio was higher in IA patients (3.42, CI 3.43–8.03) with cardiovascular events the prominent cause of death in both groups. RA is as frequent in the IA population of Australia as in the NI population. However, IA patients with RA present at younger age, have similar rates of extraarticular manifestations, but receive less rheumatological care and experience higher mortality.

**Supplementary Information:**

The online version contains supplementary material available at 10.1007/s00296-025-05951-y.

## Introduction

Rheumatoid arthritis (RA) is a common chronic systemic autoimmune disease affecting 0.5–1% of the global population [1, 2]. There is however limited information on disease prevalence in minority populations with reports from North and South America suggesting that RA is often more frequent and associated with more severe outcomes in Indigenous populations [3–7]. The Indigenous Australian (IA) population makes up 3% of the total population and as a group are disadvantaged relative to other Australians. They face lower levels of education, employment and income in combination with higher levels of smoking, obesity and injury resulting in lower life expectancy; this is attributed to persisting consequences of colonisation [8, 9]. Following an initial query in the 90’s on whether RA was actually occurring in the IA population [10], two very small case series have been reported over the last decades describing only six confirmed cases [11, 12]. Two recent literature reviews emphasized the virtual absence of data on RA in the IA population [13, 14]. To help address this knowledge gap, we investigated disease incidence, morbidity and mortality in a long-term cohort study of IA and non-Indigenous (NI) patients with RA.

## Methods

We included residents of Western Australia (WA), who were over the age of 15 years of age and received a recorded hospital discharge diagnosis of RA (International Classification of Diseases codes 714.x, M05.x and M06.x) in period January 1985 to December 2014. This method has 80–90% predictive value for case finding in administrative databases [15].

### Data sources

We obtained routinely collected patient specific longitudinal health data registered in the Western Australia Rheumatic Disease Epidemiological Registry (WARDER) as previously described [16, 17]. This dataset includes all discharge diagnoses and procedures for each hospital contact in individual patients as well as ED visits and time and cause of death. According to Australian Institute of Health and Welfare recommendations, IA status in WA is self-reported at health contacts. We classified individuals as IA patients if they identified as IA in more than 25% of all health contacts. The index date was the first hospital admission with a recorded RA diagnosis and inclusion ended at time of death or study end point (1.1.2015).

### Outcomes

The main outcomes were RA incidence rate per 100.000 persons (IR) based on Australian Bureau of Statistics (ABS) gender and IA/NI specific population data for WA from the 2001 census (middle population for this study). A point prevalence (PP) on 1.1.2015 (end point of this study) per 100,000 persons was based on WA specific population data from the 2016 census (available at: https://www.abs.gov.au/census/find-census-data). Other outcomes were comorbidity accrual by modified Charlson Comorbidity Index (m-CCI, which excludes the rheumatic disease score), RA readmission rates (any admission requiring RA management), subsequent need for joint surgery and confirmed (i.e. culture positive) infections (see Supplemental Table 1 for algorithms for these definitions). Survival time was the period between index date and death or end of observation. Mortality rates (MR) were based on observed numbers of death divided by total years of follow-up with index age adjusted standardised mortality rates (SMR) reflecting MR in each group versus MR for the population-based age group in WA over the study period. Causes of death were extracted from the WA Death Register that contains complete information on the date of death and also details the primary and underlying causes of death. Specific causes of death were categorised into broad categories by organ/disease corresponding to ICD categories.

The Human Research Ethics Committee at the WA Department of Health (WADOH HREC # 2016.24, April 2016) provided approval for the use of deidentified data and waived the need for informed consent. The WARDER database has been used widely to report on epidemiology and outcomes of various autoimmune conditions [16, 18].

### Statistical analysis

Descriptive statistics include median and interquartile range (IQR) for continuous variables with Kruskal-Wallis test for comparison, categorical data described as frequency and proportion and group comparison assessed with Odds Ratios (OR) and Rate ratios (RR) with 95% confidence intervals (CI) and Fisher’s exact test. Primary outcomes (IR and PP) are presented with 95% CI derived from Poisson distribution with changes in IR over time assessed by linear least squares regression analysis using the coefficient of determination (R-squared, R^2^) as the goodness-of-fit measure with high R^2^ coefficients (range 0–1) indicating a good fit for rates over time and p-values were derived from analysis of variance. Crude mortality rates (MR) are given per 100 person years for RA patients and compared between subgroups by MR ratio, both with 95% CI. The age adjusted standardised mortality rate was estimated by dividing the observed MR for subgroups by the MR for the relevant age group in the WA population over the study period as provided by Australian Bureau of Statistics (ABS) (https://dbr.abs.gov.au/region.html?lyr=ste&rgn=5). All statistics were derived using SPSS software v29.0 (IBM, USA) and OpenEpi software with two-sided p-values (p) < 0.05 considered statistically significant.

## Results

**Fig. 1:**
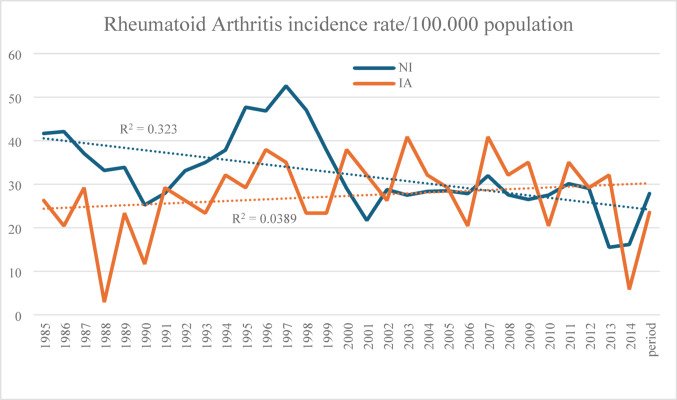
Annual incidence rate per 100.000 population for hopsitalised patients with Rheumatoid Arthritis (RA) in Indigenous (IA) and non-Indigenous Australians (NI) in Western Australia over the study period (1985–2014)

Among 14,041 patients hospitalised with RA, 282 (2%) identified as IA over the study period. Based on these figures the average IR for RA over the study period was 23.52 (CI 14.99–35.14) for IA and 27.85 (CI 18.48–40.29) for NI patients (IR ratio 0.85 CI 0.49–1.46, *p* = 0.67). Over time there was a moderate decrease in the IR for NI patients and a nonsignificant increase for IA patients (Fig. 1). With 7303 NI and 189 IA surviving patients, the point prevalence rate per 100,000 on 1/1/2015 was 373.5 (CI 336.5–413.3) for IA and 375.3 (CI 338.3–145.2) for NI patients (RR 0.99, CI 0.86–1.15) (*p* = 0.91). Demographic data demonstrated no gender differences between IA and NI patients, but a significantly lower age at index admission (42 vs. 65 years, *p* < 0.01) in IA patients, who received more care in regional hospitals, resided more in social disadvantaged areas and had accrued more comorbid conditions (Table 1).

**Table 1:** Incidence and prevalence rates per 100.000 population and patient characteristics at index date for Indigenous (IA) and non-Indigenous Australians (NI) patients with rheumatoid arthritis (RA)

	Total nr Rheumatoid Arthritis patients (*n* = 14041)		P value
	IA (*n* = 282)	NI (*n* = 13759)	
Middle population 2001 census	34,260	1,411,393	
Averaged incidence rate	23.52 (14.99–35.14 )	27.85 (18.48–40.29)	0.67
Point prevalence per 1.1.2015	373.5 (336.5-413.3)	375.3 (338.3–145.2)	0.91
Index age (IQR)	42	65	< 0.01
Female sex	203 (72)	9284 (67.5)	0.23
*Main hospital (%)*			
Regional	156 (55.2)	1954 (14.2)	
Metropolitan	126 (44.8)	11,805 (85.8)	< 0.01
*Socio-economic status (%)*			
Lowest quintile	165 (1.2)	40 (14.2)	< 0.01
Highest quintile	3219 (23.4)	49 (17.4)	
Privately insured (%)	?	?	
Comorbid conditions*			
Smoking	182 (68.2)	5172 (44.1)	< 0.01
Diabetes mellitus	108 (40.4)	1960 (16.7)	< 0.01
Obesity	64 (24.0)	1467 (12.5)	< 0.01
Hypertension	69 (25.8)	3602 (30.7)	0.21
m-CCI score	0.00 (0–1)	1.00 (0–1)	0.01
m-CCI score ≥ 3	2369 (20.2)	60 (22.5)	0.36

*Comorbidity estimates are based 267 IA and 11,739 NI patients with prior hospitalisation. m-CCI refers to modified Charlson Comorbidity Index (i.e. without rheumatic disease score)

During a total 121.151 person years of follow-up (mean 9.33, median 7.67 years) IA patients had significantly lower odds (OR 0.47,CI 0.43–0.52) and rates (13.95 vs. 29.53, RR 0.47, CI 0.43–0.52), *p* < 0.001) for an RA related readmission than NI patients (Table 2). Arthrocentesis was performed and pulse steroid therapy given as often in IA as NI patients, but synovectomy, arthrodesis and arthroplasty were significantly less frequently performed in IA patients (all *p* < 0.001). Despite lower rates of confirmed osteoporosis (0.4 vs. 4.5%), IA patients had proportionally (34.9 vs. 24,4, *p* < 0.01) more overall fractures (any type), but spinal fracture frequency (2.5 vs. 4.8%) was not significantly different from NI patients. Extraarticular RA manifestations were seen as frequent in both groups (Table 2), but IA patients were more prone to comorbidity development such as chronic kidney disease (OR 2.57; CI 1.32–4.61, *p* < 0.01) and infections (OR 2.33; 1.64–3.32, *p* < 0.01) with cardiovascular disease as frequent as in NI patients (Table 2).

**Table 2:** Disease specific and other clinical complications after index date for Indigenous (IA) and non-Indigenous Australians (NI) patients with rheumatoid arthritis (RA)

	IA (*n* = 282)	NI (*n* = 13759)	OR/RR	*P* value
Follow-up (yrs)	8.8 (3.8–14.7)	7.6 (3.1–14.4)		0.05
Total person years	2810	118,341		
Nr re-admitted for RA	94 (33.3)	7021 (51.0)	0.48 (0.37–0.62)	< 0.001
RA readmission rate	13.95 (12.60–15.41)	29.53 (29.22–29.84)	0.47 (0.43–0.52)	< 0.001
Nr ever treated with iv MP	16 (5.8)	1060 (8.3)	0.72 (0.43–1.19)	0.20
Ever joint procedure performed	72 (25.5)	5772 (41.9)	0.47 (0.36–0.62)	< 0.001
Nr with arthrocentesis	58 (20.6)	2540 (18.5)	1.14 (0.85–1.53)	0.37
Arthrocentesis	4.56 (3.8–5.42)	4.49 (4.37–4.61)	1.01 (0.85–1.20)	0.88
Nr with synovectomy infections	6 (2.1)	880 (6.4)	0.32 (0.14–0.72)	0.001
Synovectomy rate	0.39 (0.19–0.70)	1.20 (1.15–1.27)	0.32 (0.18–0.58)	< 0.001
Nr with arthrodesis infections	< 5 (1.1)	695 (9.4)	0.20 (0.06–0.63)	< 0.001
Arthrodesis rate	0.11 (0.02–0.3)	0.82 (0.77–0.87)	0.13 (0.04–0.41)	< 0.001
Nr with arthroplasty infections	21 (7.6)	2878 (20.9)	0.30 (0.19–0.48)	< 0.001
Arthroplasty rate /100 PY	1.42 (1.02–1.94)	4.32 (4.2–4.44)	0.33 (0.24–0.45)	< 0.001
*Nr with RA complications*				
Osteoporosis	< 5 (0.4)	572 (4.5)	0.08 (0.01–0.57)	< 0.001
Fractures (any)	97 (34.9)	3104 (24.4)	1.80 (1.40–2.30)	< 0.001
Spinal fractures	7 (2.5)	615 (4.8)	0.54 (0.26–1.16)	0.09
Rheumatoid nodule	< 5 (0.3)	221 (1.6)	0.22 (0.011–1.09)	0.07
Pleuritis	14 (4.9)	748 (5.4)	0.91 (0.51–1.52)	0.75
Pericarditis	< 5 (1.1)	84 (0.6)	1.75 (0.44–4.94)	0.35
Scleritis	< 5 (1.1)	71 (0.5)	2.07 (0.5–5.88)	0.24
Rheumatoid vasculitis	0 (0)	54 (0.4)	0.91(0.04–4.69	0.96
Rheumatoid lung disease	< 5 (1.1)	253 (1.8)	0.57 (0.14–1.59)	0.35
*Nr with other comorbidity*				
CVE (MI + CVA)	72 (25.5)	3713 (27)	0.93 (0.73–1.17)	0.59
CKD (stage 3–5)	11 (3.9)	214 (1.6)	2.57 (1.32–4.61)	< 0.001
Confirmed infection	37 (13.1)	836 (6.1)	2.33 (1.64–3.32)	< 0.001

Figures are numbers (%), median (IQR) and rates per 100 person years (PY). OR indicates odds ratio and RR indicates rate ratio, both with 95% CI

Small numbers given as < 5 per HREC recommendations to prevent identification

*CVE* cardiovascular event, *MI* Myocardial infarction, *CVA* cerebrovascular event, *CKD* chronic kidney disease

**Fig. 2:**
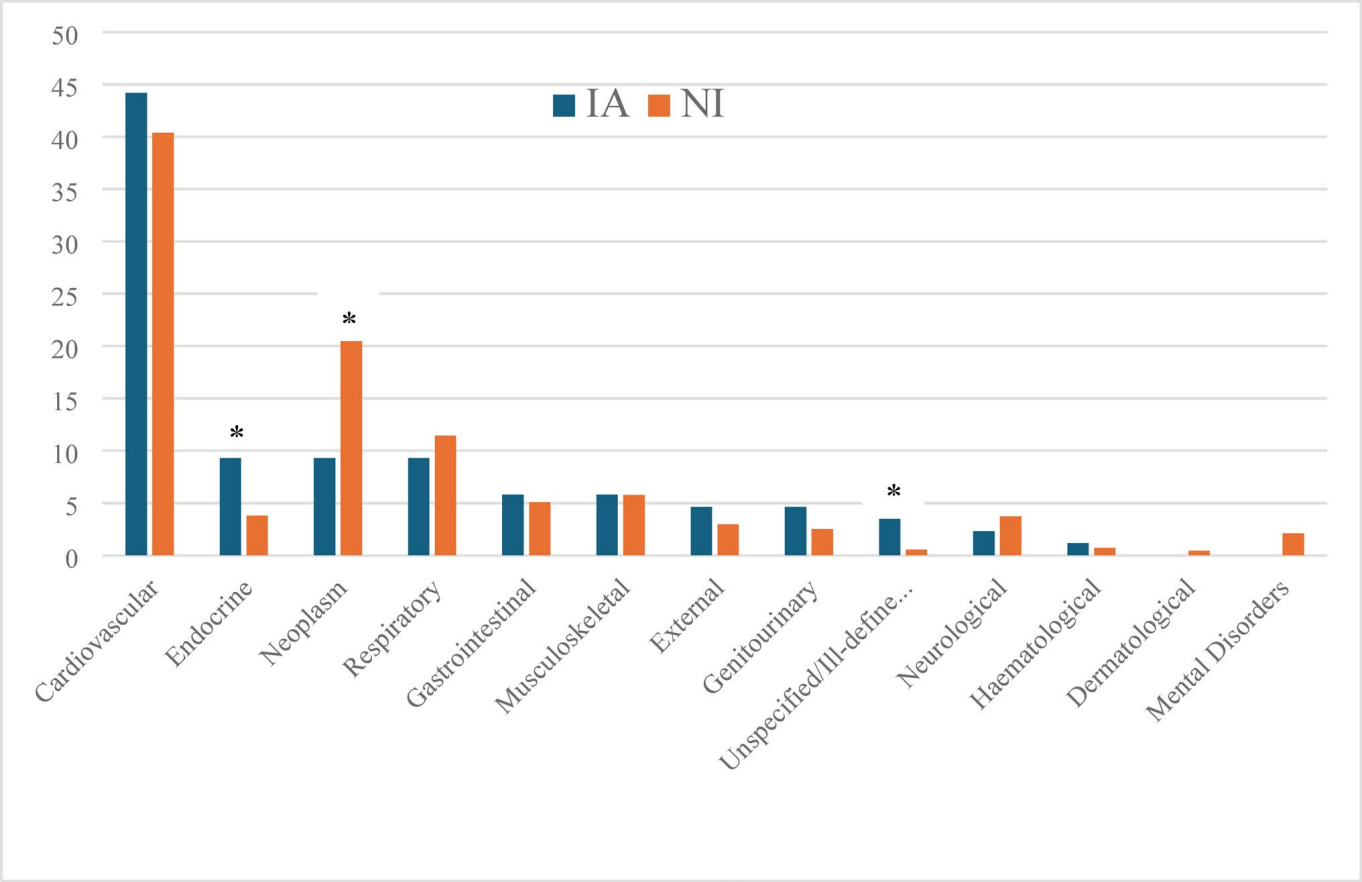
Distribution of main causes of death categories for Indigenous (IA) and non-Indigenous Australians (NI) with Rheumatoid Arthritis (RA). Bars indicate percentage of all direct causes of death. Asterix indicates a significant proportional difference between groups (*p* < 0.05)

Overall, IA patients had 2.6 times higher readmission rates for any cause (*p* < 0.001) including 1.6 times higher readmission rates for infections, while the all-cause ED visit rate was 4.7 times higher in IA patients (Table 3). RA management was the reason for ED attendance in only 0.2% of all visits for IA and 0.9% of all ED visits for NI patients (all p values < 0.001). mCCI scores increased equally in both groups and the proportion of patients with multimorbidity (mCCI > 3) at last observation did not differ. Although IA patients had lower crude mortality (OR 0.56, CI 0.43–0.72) and mortality rates (MRR 0.61, CI 0.49–0.75), the index age adjusted standardised mortality was higher in IA patients (3.42, CI 3.43–8.03) (Table 3). Causes of death analysis (Fig. 2) showed similar proportion of deaths caused by cardiovascular events for IA patients as in NI patients, with a higher proportion of death due to metabolic disease and a lower proportion of cancer deaths.

**Table 3:** General health outcomes after index date for Indigenous (IA) and non-Indigenous Australians (NI) patients with rheumatoid arthritis (RA)

	IA (*n* = 282)	NI (*n* = 13759)	OR/RR	*P* value
Follow-up (yrs)	8.8 (3.8–14.7)	7.6 (3.1–14.4)		0.05
Total person years	2810	118,341		
Nr with any re-admission readmission	264 (93.6)	12,684 (92.2)	1.24 (0.77–2.01)	0.38
Total nr re-admissions	9926	184,233		
Re-admission rate	353.2 (346.3–360.3)	155.7 (155.0–157.4)	2.27 (2.22–3.32)	< 0.001
Total nr ED visits	5757	52,027		
All cause ED visit rate	204.9 (199.61–210.23)	43.96 (43.59–44.34)	4.66(4.54–4.79)	< 0.001
ED visit for RA	14 (0.2)	466 (0.9)	0.25 (0.15–0.44)	< 0.001
m-CCI score accrual (median -IQR)	3 (2–6)	3 (1–5)		0.10
m-CCI score accrual > 3	133 (47.2)	5782 (41.6)	1.24 (0.98–1.57)	0.24
Non-survivors	93 (33.0)	6452 (46.9)	0.56 (0.43–0.72)	< 0.001
Mortality rate	33.1 (26.7–40.5)	54.5 (53.2–55.9)	0.61 (0.49–0.75)	< 0.01
MR 40–44 yrs in WA period	1.42 (1.39–1.46)	–?	–	–
MR 60–64 yrs in WA period	–?	8.01 (7.89–9.12)	–	–
Age adjusted SMR	23.31 (19.0–28.61)	6.81 (6.62–7.02)	3.42 (3.43–8.03)	< 0.01

Figures are numbers (%), median (IQR) and rates per 100 person years. OR indicates odds ratio and RR indicates rate ratio, both with 95% CI

m-CCI refers to modified Charlson Comorbidity Index (i.e. without rheumatic disease score)

*ED* Emergency Department, *MR* mortality rate, *SMR* standardised mortality rate

## Discussion

This population-based cohort study is the first to describe the clinical epidemiology of RA in Indigenous Australians. We found similar incidence and point prevalence of RA in IA and NI populations, with IA patients much younger at index diagnosis. Over the disease course, IA patients had lower readmission and ED visits rates for their underlying RA, were less likely to undergo joint surgery and had lower rates of confirmed osteoporosis, despite higher overall fracture rates. The age adjusted SMR was 3.5 times higher in IA patients with RA with cardiovascular events the most prominent cause of death.

There are no paleopathological indications to support the occurrence of RA in IA before European settlement in Australia and evidence of RA in IA has remained sparse with fewer than 100 patients described over the last 30 years in small case series [10, 12–14]. The majority of studies on RA in indigenous populations has come from North America, where RA prevalence in Haida nations and Inuit People is similar to the NI population in Canada, while in contrast the Cree People of Canada, the Pima and Chippewa People of the central US regions, and the Tlingit People of Alaska have a very high estimated RA prevalence at 2–3% [7, 19–21]. Studies from Oceania have reported a similarly widely diverging RA prevalence ranging from 0 to 3.3% in three different Māori tribes in New Zealand [7]. This study demonstrates that in Australia the incidence of RA in IA closely mirrors that of RA in NI. This is in line with some of the studies above and with results from a recent systemic review that found studies using an appropriate population comparator showed a similar risk for RA in rural and remote populations [22]. Importantly, the RA prevalence in IA of 0.38% is significantly lower than the often quoted 2% self-reported RA rate reported by the Australian Institute of Health and Welfare (AIHW) [8].

Notably, the index age of RA was much lower in IA compared to NI, which has been a consistent finding in other Indigenous populations [3, 7, 21]. Although this is not fully understood, early age at first pregnancy has been suggested as a potential explanation, given the increased risk of RA onset in the postpartum period [23]. While shared rheumatoid epitope (SE) earlier was reported to occur infrequently in IA in a small series of cases 4/7 IA patients with RA carried SE [11] and 25% of IA patients with renal disease in the tropical Kimberley region of WA carried HLA-DRB1*1409 [24]. Taken together, this indicates that a combination of genetic admixture and lifestyle (e.g. early pregnancy, smoking) likely impact disease predilection for RA in IA [10, 25] and more importantly that RA no longer should be considered a rare condition in IA in Australia [10].

In line with sparse epidemiological data, there is very limited data on disease severity of RA in IA [22]. The earliest paper on RA in IA described erosive disease and rheumatoid nodules in the majority of seven patients from Northern Australia [11], but more recent data on disease severity are lacking. While our dataset lacked clinical details, chronic inflammation in RA can drive erosive joint damage and a range of extra articular manifestations. The proportion of patients developing extra articular manifestations (rheumatoid nodules, serositis, scleritis, rheumatoid lung disease) was in line with existing literature [26, 27] and showed little difference between IA and NI patients. This together with similar numbers of patients undergoing arthrocentesis and requiring pulse MP treatment for disease flares confirms recent data that such disease complications occur similarly in metropolitan and remote populations of RA patients [22]. On the other hand, bone density loss was less frequently documented in IA patients despite a higher proportion sustaining fractures (including spinal) and there were lower odds and rates for RA related readmission and joint surgery in IA patients. This suggests a reduced ability to access rheumatology health care services and bone health screening for IA patients, as these are less available in rural health clinics than metropolitan areas [28–30]. Alternatively, this could also indicate a lesser willingness to access Western style medical care for RA or the possibility of less erosive disease in IA patients [28, 31].

Despite a much lower index age for IA patients, cardiovascular events were as frequent in both patient groups and a higher proportion of IA patients developed CKD. This accrual of multimorbidity in IA patients has long been recognised as a reason for health disparity for the IA population in general [32]. While grounded in higher rates of diabetes mellitus (DM), obesity and smoking [32] which are also reflected in our dataset, such multimorbidity is unlikely to be a direct result of underlying RA, even though various aspects of RA may contribute to it. This multimorbidity also explains the overall higher rates for any cause readmission and ED visits and has been described as a significant contributor to early mortality in IA patients [32]. The finding of higher age adjusted mortality in IA patients with RA is in keeping with this trend and also in line with the increased mortality reported in North American Indigenous RA populations [4]. Most deaths were due to cardiovascular events, which have been shown to occur at younger age in the IA population [33], while metabolic events (DM) were a more frequent and neoplasm a less frequent cause of death than in NI patients with RA.

The strengths of this study lies in its ability to provide first ever population data through the largest cohort of IA patients with RA described to date, the use of a matched comparator group and analysing well validated outcomes over a lengthy period of follow-up. The limitations to this study include the use of statewide administrative health data. In this system, all physicians’ written discharge diagnoses are converted to ICD codes by qualified clinical coders, but the database lacks results from imaging and laboratory investigations. Also, data on medication (e.g. DMARD) are not available as they fall under the responsibility of the federal government. Thus, we could not study these aspects for more in-depth analysis of disease severity or their impact on prognosis. The WARDER database, established in 2017 includes health data collected up to 2015, but changes to disease frequency and severity for IA patients may have occurred since. Finally, despite the long inclusion period we cannot exclude that we missed RA patients never needing any form of hospital care and the reported incidence and prevalence rates must be considered minimum data for that period.

In conclusion, RA was as frequent in the IA as in the NI population of WA over a 30-year period but presented at a much younger age. While RA severity in terms of extra articular manifestations was similar, IA patients received less rheumatology-based health care and experienced early accrual of multimorbidity, that contributed to increased cardiovascular mortality. Resolving this imbalance by improving rheumatological health care provision is the best way forward to close this gap for Indigenous Australian patients with RA.

## Supplementary Information

Below is the link to the electronic supplementary material.

Supplementary Material 1

## Data Availability

The data that support the findings of this study were used under license from Western Australian Data Linkage Services. Restrictions apply to the availability of these data, but upon reasonable request and following permission of WA Health and Western Australian Data Linkage Services, data are available from the authors.

## Declarations

### Editing support/AI

No part of the revised manuscript contains copied parts and there was no use of external editing support or AI.

### Data statements

Approval was obtained from the Human Research Ethics Committee at the WA Department of Health (WADOH HREC# 2016.24). As this study was considered negligible risk by the WA Health HREC and due to the de-identified nature of the linked health data set, the requirement for patient consent was waived. WA Health is proprietor of this administrative health data dataset.
