# Supplementary material for: Epidemiology and complications of rheumatoid arthritis in the Indigenous Australian population
Source: Rheumatol Int. 2025 Aug 7;45(8):185. doi: 10.1007/s00296-025-05951-y (PMC12331860; doi:10.1007/s00296-025-05951-y)
Supplement: Supplementary file 1 — Supplementary Material 1 [file 296_2025_5951_MOESM1_ESM.docx]

**Supplementary Table 1- List of diagnostic and procedure codes used**

|  | **ICD-9-CM diagnosis and procedures** | **ICD-10-AM diagnosis and ACHI procedures** |
| --- | --- | --- |
| Rheumatoid Arthritis | 714.x | M05.x-M06.x |
| Interstitial lung disease | 515, 516.3, 516.8, 516.9 | J84.1, J84.8, J84.9 |
| Smoking (ever) | 305.1, V15.82 | F17, Z72.0, Z86.43 |
| Hyperlipemia | 272.x | E78.x |
| Diabetes Mellitus | 250 | E10-E14 |
| Obesity | 278.0, 278.1 | E65.x, E66.x |
| Pleuritis | 420.0-9,511.1,511.8,511.9 | I30.0-I32,J90,J91.8,J94.8 |
| Arthroplasty | 81.51-59, 81.70-75,81.80-88 | 46300-00 to 46324.00, 46321.00 to 46324.00, 47522-00, 47522.0049717-00,50127.00.90537-00.90543-00 |
| Arthrodesis | 81.0-81.39 | 45877-00 to 46303-00, 35400-00, 40331-00,48939-00,48941-00, 49106-00 to 49845-00, 90016-00,90559-00 |
| Synovectomy | 80.70-80-79 | 46336-0046360-00, 49954-00 to 49860-00, 50311-00 to 53116-00 |
| Arthroscopy | 80.10-80.19 | 45855-00 to 45857-01, 8945-00 to 48960.00 , 49109-00 to 49117-00, 49360-0049700-00, 50100-00 to 50103-00 |
| Arthrocentesis | 81.90-81.97 | 49303-00 to 49706-01, 50100-00 to 50124-01, |
|  |  |  |
|  |  |  |
| Codes used to calculate CCI/m-CCI | Quan 2005 , Nossent 2024 | |
|  |  | |
